# The HEAVEN criteria predict laryngoscopic view and intubation success for both direct and video laryngoscopy: a cohort analysis

**DOI:** 10.1186/s13049-019-0614-6

**Published:** 2019-04-24

**Authors:** Fauzia Nausheen, Nichole P. Niknafs, Derek J. MacLean, David J. Olvera, Allen C. Wolfe, Troy W. Pennington, Daniel P. Davis

**Affiliations:** 1 Department of Medical Education, California University of Science & Medicine, School of Medicine, 217 E Club Center Dr Suite A, San Bernardino, CA 92408 USA; 2 Department of Emergency Medicine, Arrowhead Regional Medical Center, Colton, USA; 3 Air Methods Corporation, Greenwood Village, Colorado USA

**Keywords:** Airway management, Intubation, Laryngoscopy, Resuscitation

## Abstract

**Background:**

Existing difficult airway prediction tools are not practical for emergency intubation and do not incorporate physiological data. The HEAVEN criteria (Hypoxaemia, Extremes of size, Anatomic challenges, Vomit/blood/fluid, Exsanguination, Neck mobility) may be more relevant for emergency rapid sequence intubation (RSI).

**Methods:**

A retrospective analysis included air medical RSI patients. A checklist was used to assess HEAVEN criteria prior to RSI, and Cormack-Lehane (CL) laryngoscopic view was recorded for the first intubation attempt. The incidence of a difficult (CL III/IV) laryngoscopic view as well as failure to intubate on first attempt with and without oxygen desaturation were determined for each of the HEAVEN criteria and total number of HEAVEN criteria. In addition, the association between HEAVEN criteria and both laryngoscopic view and intubation performance were quantified using multivariate logistic regression for direct laryngoscopy (DL) and video laryngoscopy (VL) configured with a Macintosh #4 non-hyperangulated blade.

**Results:**

A total of 5137 RSI patients over 24 months were included. Overall intubation success was 97%. A CL III/IV laryngoscopic view was reported in 25% of DL attempts and 15% of VL attempts. Each of the HEAVEN criteria and total number of HEAVEN criteria were associated with both CL III/IV laryngoscopic view and failure to intubate on the first attempt with and without oxygen desaturation for both DL and VL. These associations persisted after adjustment for multiple co-variables including the other HEAVEN criteria.

**Conclusion:**

The HEAVEN criteria may be useful to predict laryngoscopic view and intubation performance for DL and VL during emergency RSI.

**Electronic supplementary material:**

The online version of this article (10.1186/s13049-019-0614-6) contains supplementary material, which is available to authorized users.

## Background

Tracheal intubation remains the gold standard for emergency airway management, providing support for oxygenation and ventilation as well as airway protection from aspiration. The use of neuromuscular blocking agents is often required to facilitate optimal visualization of glottic structures and increase intubation success. However, the resultant apnea may result in oxygen desaturation, which can increase morbidity and mortality [1–3] .

Predicting a difficult airway is important to assist with risk-benefit analysis and to guide the optimal approach to airway management. Difficult airway assessment tools, such as Mallampati or LEMON [Look externally, Evaluate 3–3-2 rule, Mallampati, Obstruction, Neck mobility] combine intuitive elements (e.g., “Look externally”) with assessments developed in the pre-operative setting that require patients to be awake and cooperative (e.g., “Evaluate 3–3-2” and “Mallampati”) [4, 5]. In addition, these do not incorporate physiological factors, which have been advocated, in combination with anatomic factors, as important predictors of the difficult airway [6]. More recently, the HEAVEN criteria have been proposed as more relevant and feasible for emergency airway assessment [7, 8].

- Hypoxemia – oxygen saturation value ≤93% at the time of initial laryngoscopy
- Extremes of size – pediatric patient (≤8 years of age) or clinical obesity, defined by the operator as anticipated to interfere with either bag-valve-mask ventilation and/or visualization of glottic structures during laryngoscopy
- Anatomic challenge – any structural abnormality that is anticipated to limit laryngoscopic view; this may include trauma to the airway structures themselves, limited oral aperture, large tongue, short neck, mass lesion or swelling, foreign body, or external structure that limits laryngoscopy or obstructs visualization
- Vomit/blood/fluid – clinically significant fluid noted in the pharynx/hypopharynx prior to laryngoscopy that is anticipated to interfere with either bag-valve-mask ventilation and/or visualization of glottic structures during laryngoscopy
- Exsanguination – suspected anemia, either chronic (based on past medical history) or acute (based on chief complaint, mechanism of injury, or examination findings), raising concerns about limiting safe apnea times.
- Neck mobility issues – limited cervical range-of-motion

Each of the HEAVEN criteria was associated with lower intubation success rates than with no criteria present, and the total number of HEAVEN criteria was inversely proportional to first attempt intubation success, overall intubation success, and first attempt intubation success without oxygen desaturation [8].

The objective of the present study is to explore the relationship between HEAVEN criteria, laryngoscopic view, and intubation failure for both direct and video laryngoscopy. We hypothesized that each of the HEAVEN criteria as well as the total number of HEAVEN criteria present would predict a greater likelihood of a difficult airway view and higher rates of intubation failure for both direct and video laryngoscopy.

## Methods

### Design

This is a retrospective analysis using the Air Methods Airway Registry, which includes data from patients treated by air medical providers from 160 bases across the United States. The study was approved by the appropriate Institutional Review Board (IRB), and the requirement for written informed consent was waived by the IRB. This manuscript adheres to the applicable STROBE guidelines.

### Setting

At the time of this analysis, Air Methods Corporation included 160 air medical bases across the United States. Air medical crews consisted of a flight nurse and flight paramedic responding to both scene and interfacility calls at the request of treating ground emergency medical service (EMS) providers or physicians. Individual providers performed an average of 2–4 field intubations per year with Air Methods but were required to document a minimum of 6 intubations per year, with additional experience from other professional positions, operating room intubations, or in the cadaver laboratory. Air medical crews received extensive education in critical care and resuscitation as part of the Helicopter Advanced Resuscitation Training (HeART) curriculum. Critical thinking was addressed through conceptual learning techniques, while cadavers, skills manikins, and high fidelity human patient simulators were used for technical and integrated training. Crew members also learned airway assessment strategies to anticipate and document airway difficulties. These included the HEAVEN criteria for difficult airway prediction as well as the Cormack-Lehane classification system for laryngoscopic view.

Air medical crews utilized RSI for all non-arrest patients requiring endotracheal intubation. Preoxygenation was performed using passive oxygenation with a non-rebreather oxygen mask and either assisted or post-paralysis ventilation using bag-valve-mask (BVM) [9]. Oxygen saturation values above 93% were targeted prior to laryngoscopy based on our previous study defining desaturation patterns [10]. Crews applied the HEAVEN difficult airway assessment tool prior to administration of RSI medications. If one or more criteria were present, alternative strategies were recommended. These included: use of external laryngeal manipulation, deep insertion of the laryngoscope blade into the esophagus followed by withdrawal until visualization of arytenoids, use of a gum-elastic bougie, or access of either a supraglottic device or cricothyrotomy kit. In addition, primary use of direct laryngoscopy was suggested for criteria that require speed (hypoxemia, exsanguination, copious amounts of vomit/blood/fluid), while video laryngoscopy was recommended for others (extremes of size, anatomic challenges, neck mobility). The specific steps involved in preparation and preoxygenation, including review of each of the HEAVEN criteria, were incorporated into a checklist that was handed to a ground EMS provider to read aloud prior to administration of RSI medications. For this analysis, use of the checklist helped to establish the presence/absence of HEAVEN criteria prior to laryngoscopy and minimize the impact of recall or reporting bias.

Etomidate, ketamine, and midazolam were available as induction agents, while succinylcholine and rocuronium were used for paralysis. High-flow apneic oxygenation (10–15 L/min) was delivered via nasal cannula during the entire RSI procedure. Air medical crews used the CMAC laryngoscope, which allowed for either direct or video-assisted laryngoscopy. The CMAC was configured with a Macintosh #4 non-hyperangulated blade for all intubation attempts. Cricoid pressure was initiated with positive-pressure ventilation and following administration of RSI medications but was discontinued if determined to interfere with ventilation. Intubators were encouraged to perform external laryngeal manipulation during laryngoscopy to facilitate and maintain optimal glottis visualization. Adjunctive devices and techniques included the gum-elastic bougie, supraglottic device, or surgical cricothyrotomy. Confirmation of tracheal positioning was performed using waveform capnography. This airway program has resulted in intubation success rates that exceed 99% [9].

All advanced airway attempts were documented by air medical crews in a protected performance improvement database, with up to 150 unique data elements collected for each patient. Data entry occurred following delivery of each patient to a receiving facility, and documentation of certain data elements (demographic information, HEAVEN criteria, intubation success) was mandatory. Regional and national performance improvement personnel reviewed each entry for completeness and accuracy, with air medical providers notified to add or correct entries as indicated within 72 h. In addition, linkage to the electronic patient care record was provided for additional data reference or verification.

### Subjects

All patients in whom a neuromuscular blocking agent was administered to facilitate tracheal intubation were included in this analysis. This excluded intubation attempts in which a paralytic was not administered, which applies primarily to arrest victims under our intubation protocols. Complete data regarding HEAVEN criteria, Cormack-Lehane laryngoscopic view, and airway management success were required for inclusion. A total of 64 patients were excluded due to missing Cormack-Lehane view documentation. A 24-month study period from December 2014 to November 2016 was defined to target approximately 1000 first attempts at intubation using direct laryngoscopy with a 10% failure rate [11].

### Statistical analysis

The intent of this analysis was to explore the relationship between the HEAVEN criteria and both Cormack-Lehane laryngoscopic view as well as intubation performance. Two outcome measures were used to define intubation performance: a) failure to place an tracheal tube on the first attempt, and b) failure to place an tracheal tube without oxygen desaturation (SpO2 < 90%). Data for direct and video laryngoscopy were presented separately given the potential differences between these techniques with regard to both laryngoscopic view and intubation performance. To facilitate the use of binomial logistic regression, Cormack-Lehane laryngoscopic view was dichotomized into “Good Laryngoscopic View” (grade I or II) and “Difficult Laryngoscopic View” (grade III or IV).

Three strategies were used to validate the HEAVEN assessment tool. First, univariate analysis was used to evaluate each of the HEAVEN criteria, comparing patients in whom each criterion was present or absent with regard to the incidence of a CL III/IV laryngoscopic view as well as intubation performance (“failure to intubate on the first attempt” and “failure to intubate on the first attempt without oxygen desaturation”) for both direct and video laryngoscopy. Chi-square analysis was used for all univariate comparisons. Second, the total number of HEAVEN criteria present was modelled against the incidence of a CL III/IV laryngoscopic view for both direct and video laryngoscopy using chi-square test-for-trend. Third, multiple logistic regression was used to explore the independent relationship between each of the HEAVEN criteria as well as the total number of HEAVEN criteria present and intubation performance (“failure to intubate on the first attempt” and “failure to intubate on the first attempt without oxygen desaturation”). Logistic regression models adjusted for the following co-variables: years of age (0–8, 9–70, 71+), sex, choice of paralytic (succinylcholine/rocuronium), location of intubation (on scene/en route), clinical category [burn, medical, neurologic, trauma, other], and individual HEAVEN criteria (except when total number of HEAVEN criteria was modelled). Logistic regression models were run separately for direct and video laryngoscopy. Appropriateness of model selection was assessed using Hosmer-Lemeshow goodness-of-fit testing. StatsDirect™ (Cheshire, UK) was used for all statistical calculations with statistical significance was assumed for a *p*-value less than 0.05 for all comparisons.

## Results

A total of 5885 patients underwent intubation attempts by air medical crews during the study period. A total of 5201 patients (88%) received neuromuscular blocking agents as part of an RSI procedure. Cormack-Lehane grades were not documented in 64 patients (1%), with an overall intubation success rate of 88% and a first-attempt success rate of 73% in these patients. This resulted in a study cohort of 5137 patients (Additional file 1: Figure S1). Cormack-Lehane grade was documented for direct laryngoscopy alone in 856 RSI patients, video laryngoscopy alone in 3614 patients, and both direct and video laryngoscopy in 667 patients. A difficult laryngoscopic view was documented in 25% of patients with direct laryngoscopy and in 15% of patients with video laryngoscopy. Tracheal intubation was ultimately successful in a total of 4970 RSI patients (97%) and was successful on the first attempt in a total of 4682 RSI patients (91%) and on the first attempt without oxygen desaturation in a total of 4582 RSI patients (89%). Demographic and clinical data for the study population as well as the 64 patients in whom Cormack-Lehane grades were not documented are displayed in Table 1.

**Table 1:** Demographic and clinical data

Parameter	DL Only (*n* = 856)	VL Only (*n* = 3614)	Both DL & VL (*n* = 667)	Neither (*n* = 64)
Demographics				
Age 0–8 years (%)	5 (4–7)	3 (3–4)	3 (1–4)	9 (2–17)
Age > 70 years (%)	14 (12–17)	15 (14–16)	13 (10–15)	17 (8–27)
Male gender (%)	66 (63–70)	68 (67–70)	70 (67–74)	80 (70–90)
Clinical Category				
Burn (%)	3 (2–4)	3 (3–4)	4 (3–5)	3 (1–11)
Medical (%)	21 (18–23)	18 (17–19)	17 (14–20)	17 (10–28)
Neurologic (%)	16 (13–18)	15 (14–16)	13 (10–15)	8 (3–17)
Other (%)	0 (0–1)	0 (0–0)	0 (0–1)	2 (0–8)
Trauma (%)	60 (57–64)	64 (62–65)	66 (62–70)	70 (58–80)
HEAVEN Criteria				
Hypoxemia (%)	9 (7–11)	9 (8–10)	10 (8–13)	9 (2–17)
Extremes of size (%)	10 (8–12)	13 (12–14)	13 (11–16)	11 (3–19)
Anatomic (%)	9 (7–11)	9 (8–10)	13 (11–16)	25 (14–36)
Vomit/blood/fluid (%)	35 (32–38)	38 (36–39)	43 (39–47)	52 (39–64)
Exsanguination (%)	1 (0–2)	1 (1–2)	3 (1–4)	3 (0–8)
Neck mobility (%)	23 (21–26)	28 (26–29)	30 (26–33)	20 (10–30)
Total # of criteria (mean)	0.9 (0.9–1.0)	1.0 (1.0–1.1)	1.2 (1.1–1.3)	1.3 (1.0–1.6)
Intubation Data				
Performed en route (%)	10 (8–12)	10 (9–11)	8 (6–11)	8 (1–15)
Succinylcholine as paralytic (%)	80 (77–82)	75 (74–77)	79 (76–82)	77 (66–87)
Overall success (%)	96 (95–98)	97 (97–98)	95 (93–96)	88 (79–96)
1st attempt success (%)	91 (89–93)	91 (90–92)	85 (82–88)	73 (62–83)
1st attempt success without oxygen desaturation (%)	90 (87–91)	89 (88–90)	83 (80–86)	64 (52–75)

Demographic and clinical data for patients with documentation of Cormack-Lehane class during direct laryngoscopy (DL) only, video laryngoscopy (VL) only, both DL and VL, or neither. All values represent mean or % (95% confidence intervals)

**Fig. 1:**
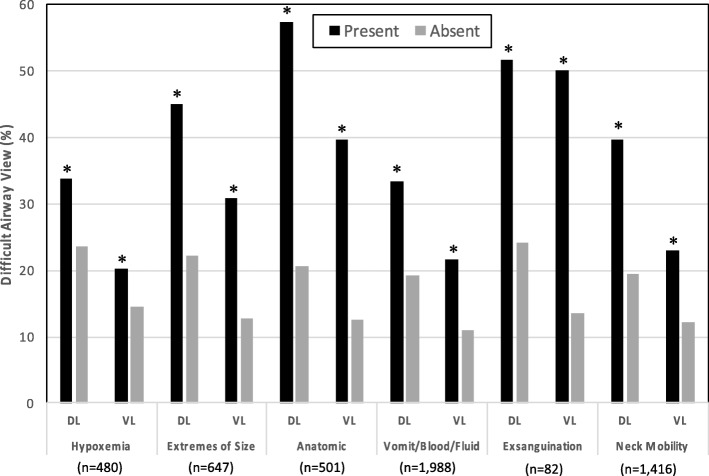
Percentage of a difficult airway view (Cormack-Lehane grade III or IV) in the presence or absence of each of the HEAVEN criteria for both direct and video laryngoscopy. Each of the HEAVEN criteria was associated with a statistically significant increase (*p < 0.01) in the likelihood of a difficult airway view. In addition, direct laryngoscopy had a higher incidence of difficult airway view for each of the HEAVEN criteria except “Exsanguination”

**Fig. 2:**
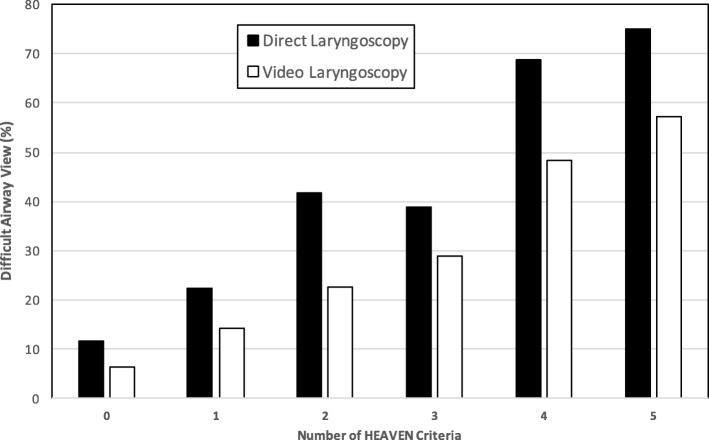
Percentage of a difficult airway view (Cormack-Lehane grade III or IV) with total number of HEAVEN criteria present. Chi-square test for trend was statistically significant for both direct and video laryngoscopy. In addition, the differences between direct and video laryngoscopy were statistically significant for 0, 1, 2, and 4 HEAVEN criteria present

**Fig. 3:**
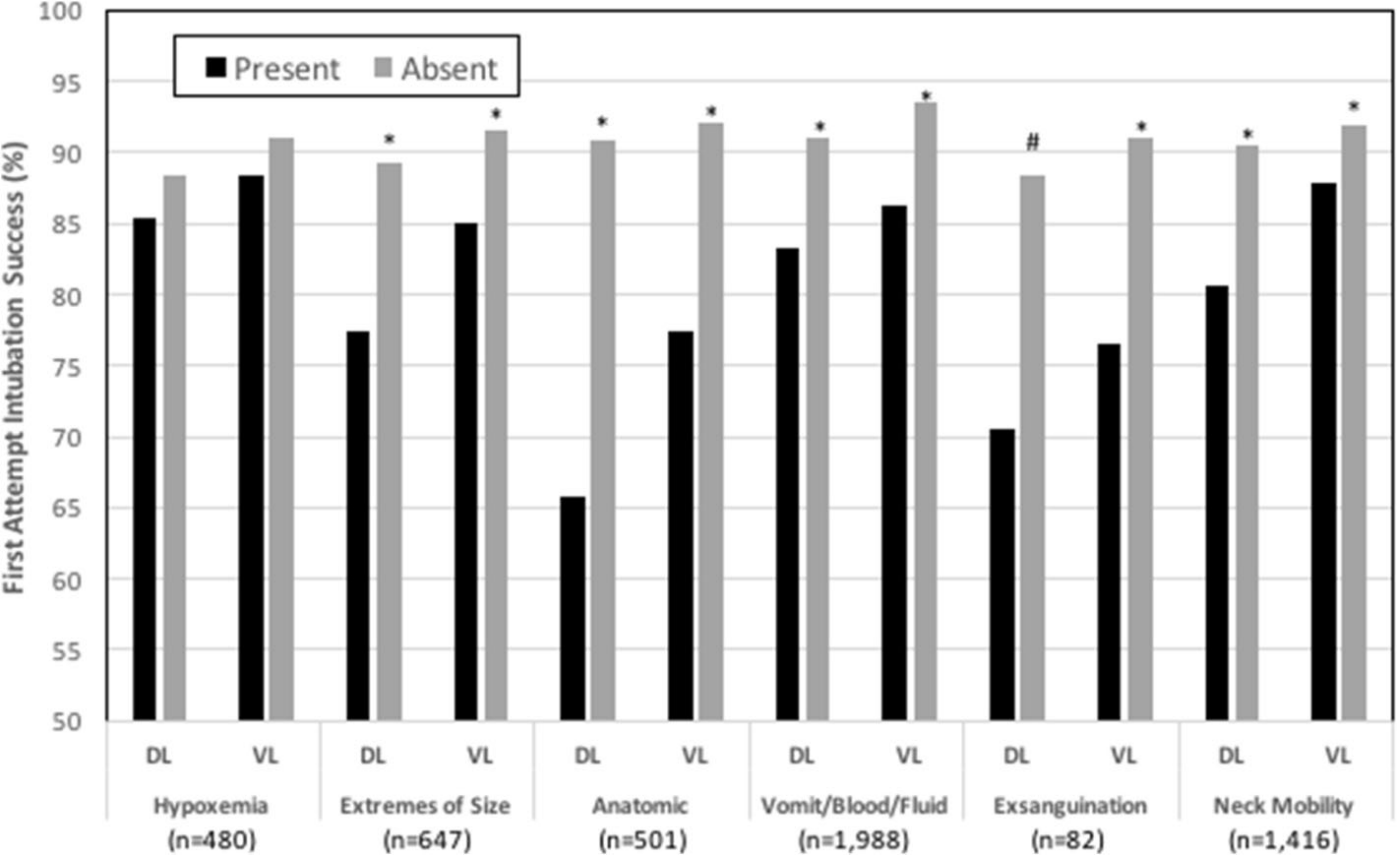
First attempt intubation success in the presence or absence of each of the HEAVEN criteria for both direct and video laryngoscopy. All comparisons between HEAVEN criteria present and absent were statistically significant (*p < 0.01) except for “Hypoxemia”

**Fig. 4:**
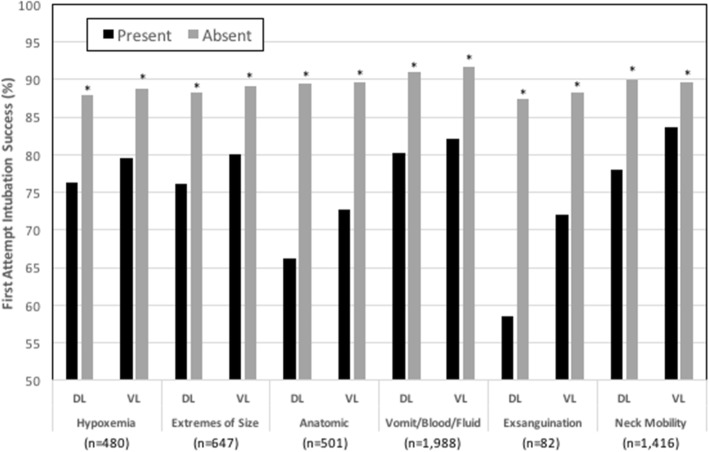
First attempt intubation success without desaturation in the presence or absence of each of the HEAVEN criteria for both direct and video laryngoscopy. All comparisons between HEAVEN criteria present and absent were statistically significant (*p < 0.01)

The percentage of patients with a difficult airway view (Cormack-Lehane grade III or IV) was statistically significantly higher in the presence of each of the HEAVEN criteria with both direct and video laryngoscopy (Fig. 1). The percentage of patients with a CL III/IV laryngoscopic view increased with a greater number of HEAVEN criteria present for both direct and video laryngoscopy (chi-square test-for-trend *p* < 0.001 for each) (Fig. 2). First attempt intubation success was lower in the presence of each of the HEAVEN criteria for both direct and video laryngoscopy except in the presence of “Hypoxemia” (Fig. 3). First attempt intubation success without oxygen desaturation was lower in the presence of each of the HEAVEN criteria for both direct and video laryngoscopy (Fig. 4).

Statistically significant independent associations were observed between individual HEAVEN criteria and failure to intubate on the first attempt, with the exception of “Hypoxemia” and “Exsanguination” (Table 2). Statistically significant independent associations were observed between each of the individual HEAVEN criteria and failure to intubate on the first attempt without desaturation (Table 2). The associations between total number of HEAVEN criteria present and both measures of intubation performance were statistically significant. Appropriate goodness-of-fit was observed for each of the models (Hosmer-Lemeshow *p* > 0.05 for all comparisons).

**Table 2:** Direct and video laryngoscopy and HEAVEN criteria to predict failure of intubation on the first attempt

Parameter	Failure at First Intubation Attempt				Failure of First Intubation Attempt without Desaturation			
	Direct Laryngoscopy		Video Laryngoscopy		Direct Laryngoscopy		Video Laryngoscopy	
	Odds Ratio (95% CI)	P	Odds Ratio (95% CI)	P	Odds Ratio (95% CI)	P	Odds Ratio (95% CI)	P
Hypoxemia	1.21 (0.81–1.83)	0.352	1.01 (0.76–1.34)	0.928	1.59 (1.01–2.51)	0.0478	1.60 (1.21–2.11)	< 0.001
Extremes of size	2.19 (1.53–3.13)	< 0.001	2.49 (1.99–3.12)	< 0.001	1.77 (1.17–2.69)	0.008	1.72 (1.34–2.21)	< 0.001
Anatomic	3.65 (2.55–5.23)	< 0.001	3.15 (2.49–4.00)	< 0.001	2.87 (1.92–4.28)	< 0.001	2.22 (1.71–2.88)	< 0.001
Vomit/blood/fluid	1.54 (1.18–2.02)	0.002	1.80 (1.50–2.17)	< 0.001	1.70 (1.21–2.38)	0.002	1.95 (1.59–2.38)	< 0.001
Exsanguination	1.74 (0.77–3.91)	0.180	1.24 (0.70–2.21)	0.456	2.48 (1.10–5.59)	0.028	1.55 (0.88–2.76)	0.132
Neck mobility	2.11 (1.76–2.21)	< 0.001	1.51 (1.24–1.84)	< 0.001	1.66 (1.17–2.35)	0.004	1.15 (0.93–1.42)	0.212
Total # criteria	1.97 (1.76–2.21)	< 0.001	1.84 (1.71–1.98)	< 0.001	1.92 (1.69–2.19)	< 0.001	1.66 (1.53–1.80)	< 0.001

Multiple logistic regression analysis exploring the relationship between the individual HEAVEN criteria or the total number of HEAVEN criteria and failure to intubate on the first attempt with and without desaturation (adjusted for age, gender, clinical category, choice of paralytic, and location of the procedure)

## Discussion

We used a large airway registry to validate a novel difficult airway prediction tool by exploring the association between the HEAVEN criteria and Cormack-Lehane laryngoscopic view as well as intubation success for both direct and video laryngoscopy. A more difficult laryngoscopic view and lower intubation success was associated with each of the HEAVEN criteria and with more HEAVEN criteria present. These associations persisted following adjustment for multiple co-variables as well as the other HEAVEN criteria themselves. This was particularly true with use of a time-dependent outcome (“failure to intubate on first attempt without oxygen desaturation”).

Predicting a difficult airway is critically important during advanced airway management, particularly with use of neuromuscular blocking agents and estimation of safe apnea time [12]. Anticipating a difficult airway assists in the decision regarding the timing and location of the procedure, personnel involved, the primary technical approach to intubation, and the availability of back-up equipment and strategies. These data support the predictive ability of HEAVEN for both laryngoscopic view as well as intubation performance.

The HEAVEN assessment tool may represent an improvement over traditional strategies [13–18]. The size of our airway database allowed us to explore multiple variables simultaneously and better validate the independent predictive ability of each of the HEAVEN criteria as well as the total number of criteria present. Other assessment strategies, such as Mallampati and LEMON, may be limited due to the impracticability of particular elements in emergent situations. In addition, HEAVEN combines anatomical data (“Extremes of size”, “Anatomical challenges”, “Vomit/blood/fluid”, “Neck mobility issues”) with physiological data (“Hypoxemia”, “Exsanguination”) as suggested by Mosier et al. [6]. This was particularly evident when using the more time-dependent outcome of “failure to intubation on the first attempt without desaturation.”

These data are consistent with our previous evaluations of the HEAVEN criteria, providing additional validation using both direct and video laryngoscopy as well as offering a potential mechanism in laryngoscopic view to explain these findings. Although comparing direct and video laryngoscopy was not the objective of this analysis, it is notable that video laryngoscopy appeared to provide superior views and intubation success for HEAVEN criteria associated with physical/structural challenges (Extremes of size, Anatomic, Vomit/blood/fluid, Neck mobility) [19–21]. This likely reflects the importance of line-of-site alignment with anatomic axes for glottic visualization with direct laryngoscopy. This differential was not as profound with HEAVEN criteria reflecting the importance of speed (Hypoxemia, Exsanguination) in safe apnea time [10, 22].

The reported incidence of difficult laryngoscopic views was higher than in other studies.^24–27^ This may reflect the critical nature of these patients, with relatively high percentages of trauma and burn patients. In addition, a higher incidence of difficult laryngoscopic views may be anticipated given the suboptimal intubation conditions in the out-of-hospital environment, including performance of the RSI procedure in the back of a moving helicopter.

Strengths of this study include the large sample size, uniform approach to airway management training and protocols, and systematic collection of data in a comprehensive airway registry. While the generalizability of emergency medical service-based studies to other settings and practitioner types may be challenged, the high intubation success rates – which have since exceeded 99% overall intubation success – underscore the relevance of these data to other advanced airway practitioners working in less extreme conditions [2, 9, 21, 22]. In addition, the HEAVEN criteria can be assessed prospectively in any setting without requirement for additional tools, devices, or patient cooperation. Nevertheless, additional investigation is necessary to validate the HEAVEN criteria in different environments and with other provider types.

Several additional limitations must be considered when interpreting these results. A certain degree of reporting and recall bias may exist with both laryngoscopic view and presence of HEAVEN criteria, since no monitoring equipment were employed to record or verify these observations. We attempted to minimize this by mandating the use of a standardized checklist, which forces providers to prospectively define the presence or absence of HEAVEN criteria prior to initial laryngoscopy. This appears to have resulted in a fairly small amount of data missingness. However, we cannot exclude the possibility that fairly to input data regarding laryngoscopic view might represent a form of selection bias since intubation success was lower in these patients.

We did not compare HEAVEN to other difficult airway assessment tools. This reflects the inability to perform key elements of difficult airway predictors such as LEMON or Mallampati. In addition, it is impractical to expect providers to perform multiple assessments when faced with critically ill and injured patients. It becomes even more difficult to compare the results presented here with prior studies evaluating alternate difficult airway assessment tools, since these were not performed during emergency RSI and were not evaluated with the rigor presented here [13, 18].

The influence of multiple confounders could not be determined. Provider type, tracheal intubation experience, patient physiologic status, airway physical characteristics, and use of cricoid pressure or other adjuncts may influence laryngoscopic view but were not included in the analysis [23–30]. The uniformity of protocols and training and the strong association between HEAVEN criteria and laryngoscope view helps to offset the influence of these factors. Ultimately, the true value of the HEAVEN criteria may best be evidenced by the improvements in intubation success over time with their use as the foundation of our advanced airway management program, with overall intubation success rates exceeding 99% [9].

Finally, the use of the term “video laryngoscopy” could be misleading given the various platforms and blade shapes currently available for clinical use. The CMAC blade used during this study is more similar to a traditional Macintosh blade, and it is possible that a more hyperangulated video laryngoscope blade might produce different results. The intent of this analysis was not to compare direct and video laryngoscopy but instead to validate the HEAVEN assessment tool. Direct and video laryngoscope data are presented separately given their anticipated impact on both laryngoscopic view as well as intubation success.

## Conclusions

The individual HEAVEN criteria as well as the total number of HEAVEN criteria present were associated with a more difficult laryngoscopic view (CL III/IV) as well as a decrease in intubation success, defined by tracheal intubation on the first attempt and on the first attempt without oxygen desaturation. These associations persisted despite adjustment for multiple covariables. These data suggest potential utility for the HEAVEN criteria as a tool for difficult airway prediction during emergency RSI. The generalizability of HEAVEN to other environments and provider types requires further investigation.

## Additional file

Additional file 1: **Figure S1.** Flow chart for study enrollment. (JPG 85 kb)
