# Supplementary figures and images for: The HEAVEN criteria predict laryngoscopic view and intubation success for both direct and video laryngoscopy: a cohort analysis
Source: Scand J Trauma Resusc Emerg Med. 2019 Apr 24;27:50. doi: 10.1186/s13049-019-0614-6 (PMC6480652; doi:10.1186/s13049-019-0614-6)

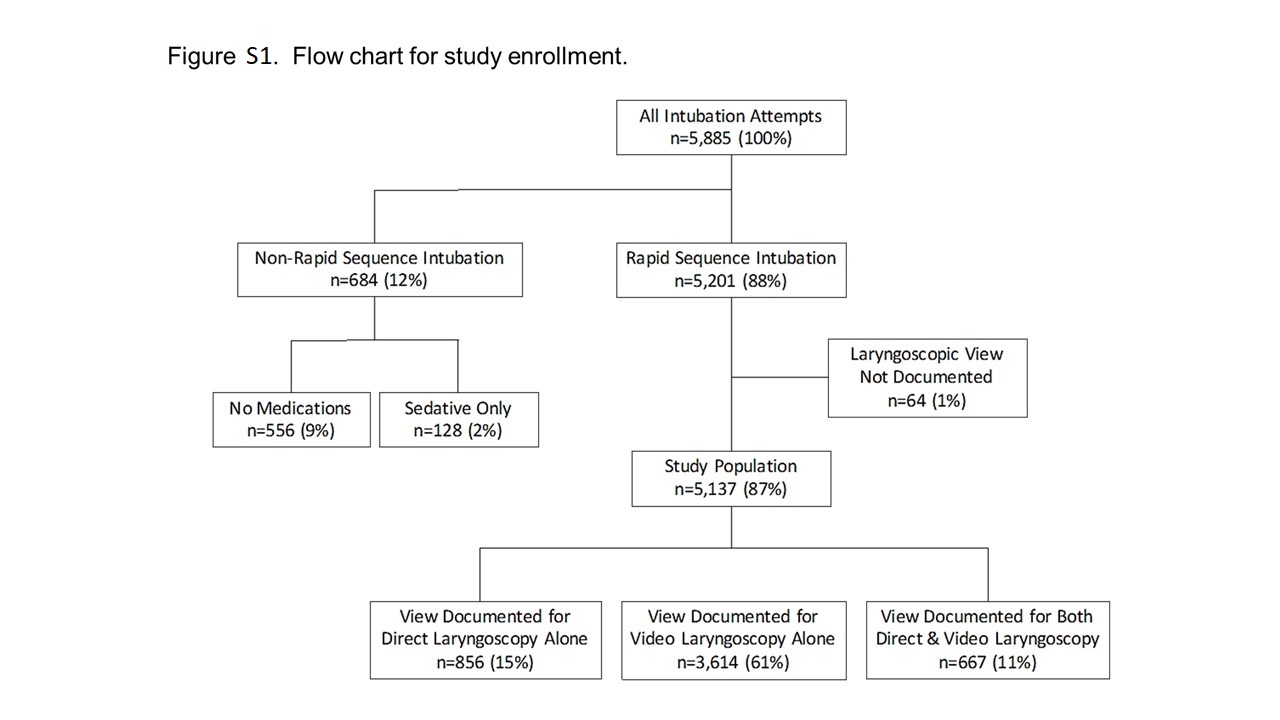

Supplement: Supplementary file 1 — Figure S1. Flow chart for study enrollment. (JPG 85 kb) [file 13049_2019_614_MOESM1_ESM.jpg]
